# The "Red Chamber" Treatment of Small-Pox Patients

**Published:** 1894-11-10

**Authors:** 


### Editor's Letter-Box.

[Our correspondents are reminded that prolixity is a great bar to publication, and that brevity of style and conciseness of statement greatly facilitate early insertion.]

### THE "RED CHAMBER" TREATMENT OF SMALL-POX PATIENTS.

Dr W. Benthall writes to us from Friargate, Derby: It may be of interest to your readers to note with reference to the treatment of small-pox patients by red light at Copenhagen in 1894, that a similar method was in vogue as long ago as the 14th century. Dr. Paris in his "Pharmacologia," published about 1820, relates that "John of Gaddesden ordered the son of Edward the First, who was dangerously ill with the small-pox, to be wrapped in scarlet cloth, as well as all those who attended him or come into his presence, and even the bed and room in which he was laid were covered with the same substance, and so completely did it answer, say the credulous historians of that day, that the prince was cured without having so much as a single mark left upon him." Dr. Paris attributes this treatment to the doctrine of signatures, and condemns it accordingly, but it is curious to see this practice renewed, upon scientific grounds, after a lapse of nearly six hundred years.

